# Efficient Adsorption and Electrochemical Detection of Cd^2+^ with a Ternary MgZnFe-Layered Double Hydroxides Engineered Porous Biochar Composite

**DOI:** 10.3390/molecules28207002

**Published:** 2023-10-10

**Authors:** Yongfang Yu, Wenting Yang, Shujuan Li, Yansha Gao, Linyu Wang, Guoqin Huang

**Affiliations:** Key Laboratory of Crop Physiology, Ecology and Genetic Breeding, School of Agriculture, Jiangxi Agricultural University, Nanchang 330045, China

**Keywords:** adsorption, electrochemical detection, MgZnFe-layered double hydroxides, porous biochar composite

## Abstract

Their unique layered structure, large specific surface area, good stability, high negative charge density between layers, and customizable composition give layered double hydroxides (LDHs) excellent adsorption and detection performance for heavy metal ions (HMIs). However, their easy aggregation and low electrical conductivity limit the practical application of untreated LDHs. In this work, a ternary MgZnFe-LDHs engineered porous biochar (MgZnFe-LDHs/PBC) heterojunction was proposed as a sensing and adsorption material for the effective detection and removal of Cd^2+^ from wastewater. The growth of MgZnFe-LDHs in the PBC pores not only reduces the accumulation of MgZnFe-LDHs, but also improves the electrical conductivity of the composite. The synergistic effect between MgZnFe-LDHs and PBC enables the composite to achieve a maximum adsorption capacity of up to 293.4 mg/g for Cd^2+^ in wastewater. Meanwhile, the MgZnFe-LDHs/PBC-based electrochemical sensor shows excellent detection performance for Cd^2+^, presenting a wide linear range (0.01 ng/L–1 mg/L), low detection limit (3.0 pg/L), good selectivity, and stability. The results indicate that MgZnFe-LDHs/PBC would be a potential material for detecting and removing Cd^2+^ from wastewater.

## 1. Introduction

Cd^2+^ is a heavy metal ion (HMI) with high and persistent toxicity, that poses a major threat to the human body and ecological health [1]. Therefore, it is of great importance to achieve its efficient detection and removal. So far, many technologies have been developed to detect HMIs, including atomic absorption spectrometry (AAS), ultraviolet spectrophotometry (UV-VIS), inductively coupled plasma (ICP), fluorescence, and electrochemical sensors [2,3,4]. Electrochemical sensing has attracted the attention of researchers because it offers irreplaceable advantages such as ease of use, ease of miniaturization, low cost, and high sensitivity, which enable the in situ detection of HMIs [5]. On the other hand, various removal technologies such as chemical precipitation, membrane separation, ultrafiltration, and adsorption, have been rapidly developed to remove HMIs [6,7]. Among these removal technologies, adsorption stands out due to its unique advantages: ease of use, remarkable adsorption effect, and wide range of applications [8]. Both the electrochemical sensing and adsorption for HMIs mainly depend on the properties of the materials used to make them function. Therefore, there is an urgent need for suitable functional materials that are inexpensive, easy to fabricate, and highly adsorptive.

Layered double hydroxides (LDHs) are well-known two-dimensional (2D) materials that possess unique properties and have received increasing attention in environmental applications, especially in the adsorption of HMIs [9]. LDHs as host–guest layered materials consist of a positively charged layer with metallic cations and charge-balanced anions in the interstices of the layer due to electrostatic attraction [10]. Their large specific surface area, high chemical stability, flexible composition, low price, and large amount of -OH on the surface make LDHs promising HMI adsorption materials [11]. However, due to their high charge density and large specific surface area, LDHs can easily accumulate, resulting in most adsorption active sites being covered, which greatly reduces their adsorption performance. To solve this problem, embedding LDHs in porous materials is an effective method to improve the agglomeration phenomenon. For example, Xu et al. [12] reported the use of etched nickel foam (NF) with a 3D open-pore structure as a carrier to facilitate the uniformly distributed NiCo-LDHs nanowire arrays. This approach effectively overcame the reunification phenomenon and exposed more adsorption sites.

Biochar (BC) is a by-product of biomass pyrolysis, which has the advantage of possessing abundant surface functional groups that are widely used for the adsorption of HMIs [13]. Moreover, the surface activity and surface structure of BC could be effectively modulated with KOH. Alkali activation could develop an ordered pore structure, abundant active sites, and a large specific surface area, which is advantageous for loading large amounts of LDHs to prevent their aggregation and effectively improve their adsorption performance [14]. On the other hand, the activated porous BC with high conductivity enables rapid electron transport [15]. Based on the above analysis, it is expected that the embedding of LDHs into the pores of BC will yield a promising bifunctional material with good adsorption and sensing properties for HMIs.

Herein, a ternary MgZnFe-LDHs engineered PBC composite was proposed for the simultaneous detection and removal of Cd^2+^ with high toxicity. In this composite system, ternary LDHs were chosen as adsorbents mainly due to their abundant adsorption sites and stronger adsorption capacity than binary LDHs. PBC was used as a carrier substance to reduce the aggregation of MgZnFe-LDHs, while improving the specific surface area, surface active functional sites, and electronic conductivity of the composite. Benefiting from the synergistic effect between MgZnFe-LDHs and porous PBC, the proposed MgZnFe-LDHs/PBC composite showed excellent electrochemical sensing of Cd^2+^ and facilitated the process of Cd^2+^ removal in aqueous solution.

## 2. Results and Discussion

### 2.1. Morphological Characterizations

**Figure 1:**
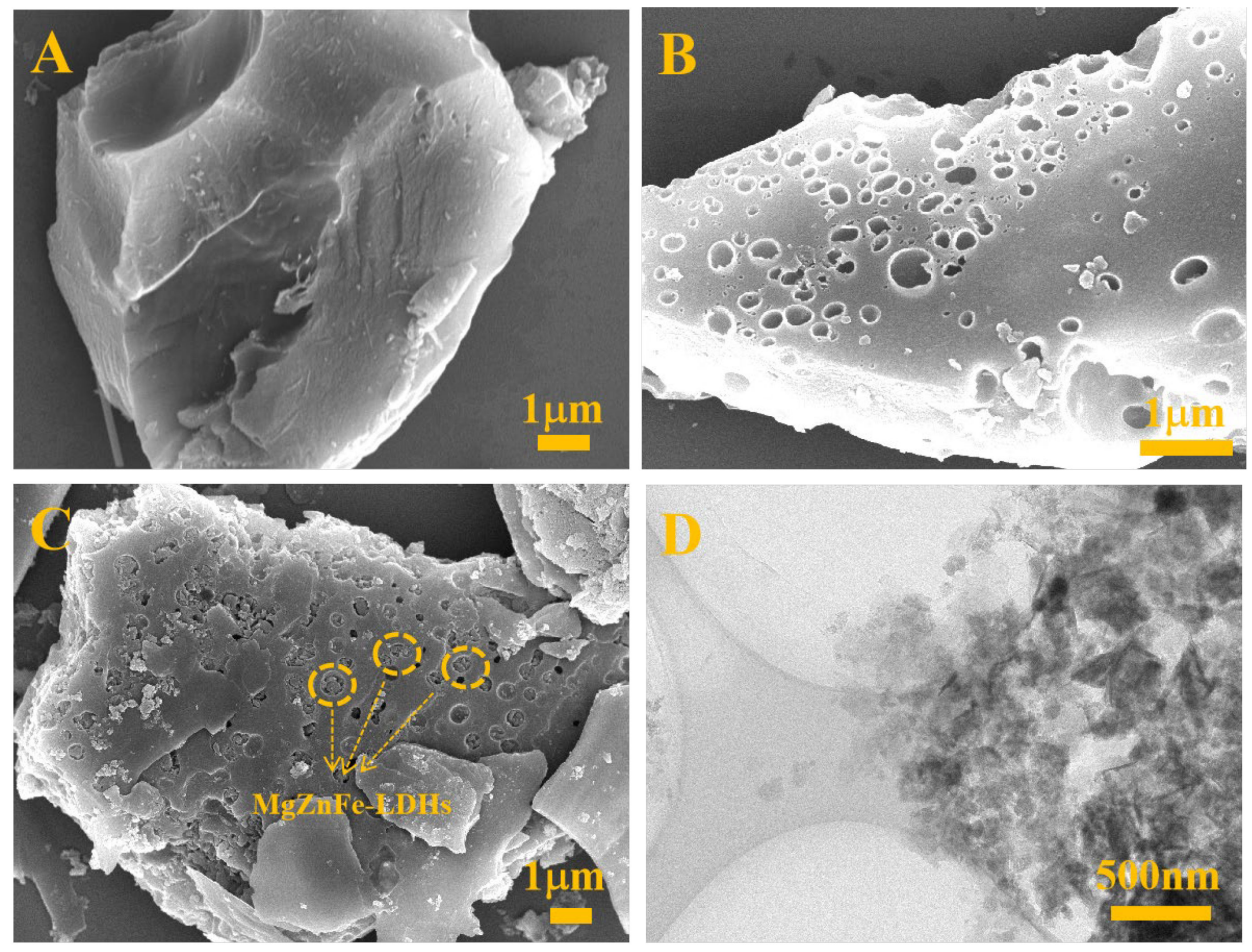
SEM of (**A**) pristine BC, (**B**) PBC, (**C**) MgZnFe-LDHs/PBC, and (**D**) TEM of MgZnFe-LDHs/PBC.

Scanning electron microscopy (SEM) of BC, MgZnFe-LDHs, and MgZnFe-LDHs/PBC was performed to study their morphology. From Figure 1A, it can be seen that the untreated BC has a massive structure with a flat surface, while a large number of pores can be observed on the PBC surface (Figure 1B), indicating that the activation process is responsible for the pore formation and effectively increases the specific surface area. For MgZnFe-LDHs/PBC (Figure 1C), it can be seen that the MgZnFe-LDHs particles are well embedded in the pores of PBC, which greatly reduces the accumulation of MgZnFe-LDHs. The transmission electron microscopy (TEM) image of MgZnFe-LDHs/PBC is shown in Figure 1D. It can be clearly seen that the MgZnFe-LDHs particles are trapped in the pores of the PBC.

**Figure 2:**
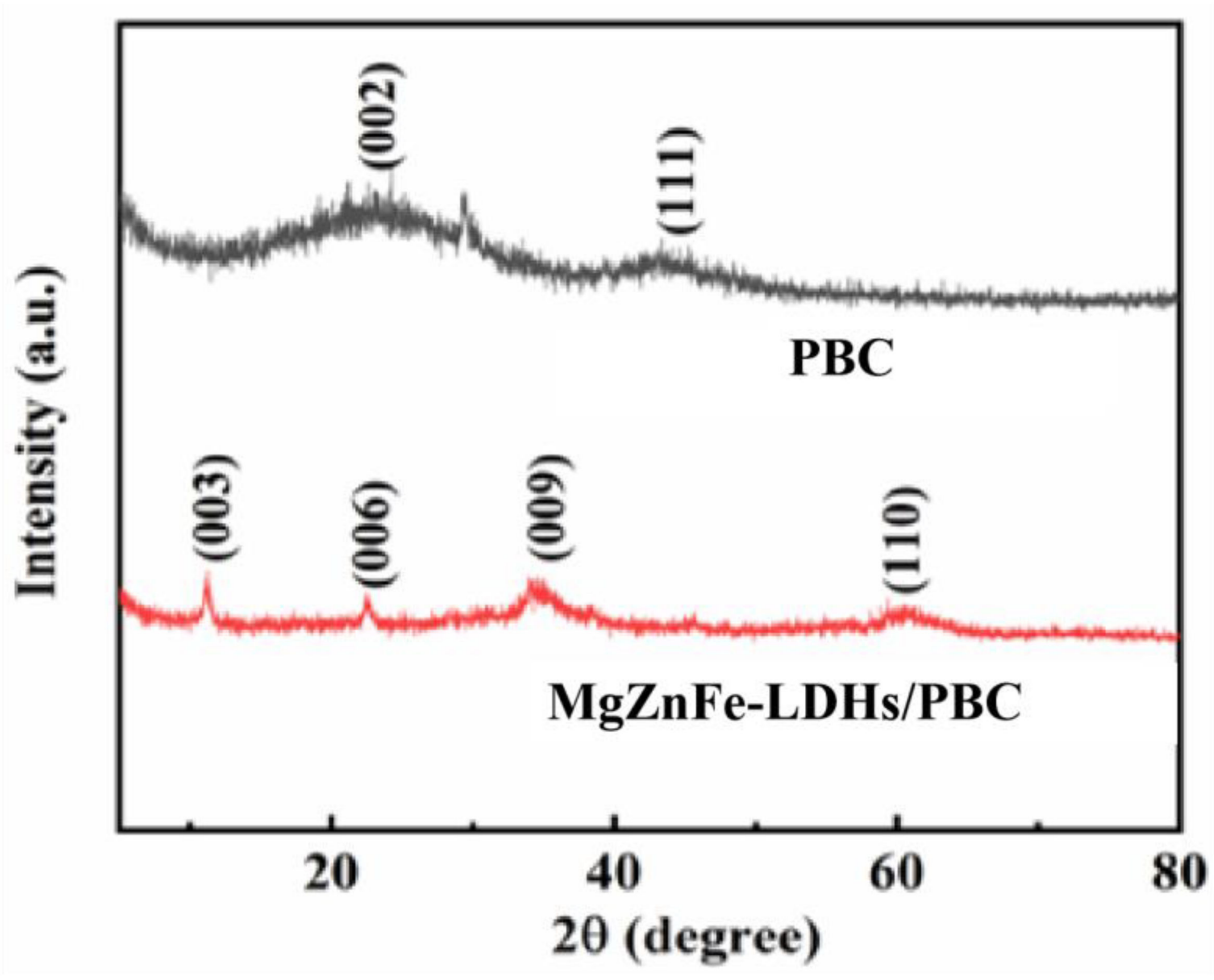
XRD patterns of PBC and MgZnFe-LDHs/PBC.

The structural characterization of BC and MgZnFe-LDHs/PBC was determined by X-ray powder diffraction (XRD). As shown in Figure 2, BC is dominated by two broadband diffraction peaks at 2θ = 22.9° and 43.5°, which can be attributed to the turbostratic carbon crystallites of biomass pyrolysis. After modification with MgZnFe-LDHs on PBC, a typical structure of the hydrotalcite compound is revealed with sharp reflection peaks at 11.6° (003), 23.4° (006), 36.3° (009), and 59.1° (110) [16]. All the above results show that the composite of MgZnFe-LDHs/PBC was successfully synthesized.

**Figure 3:**
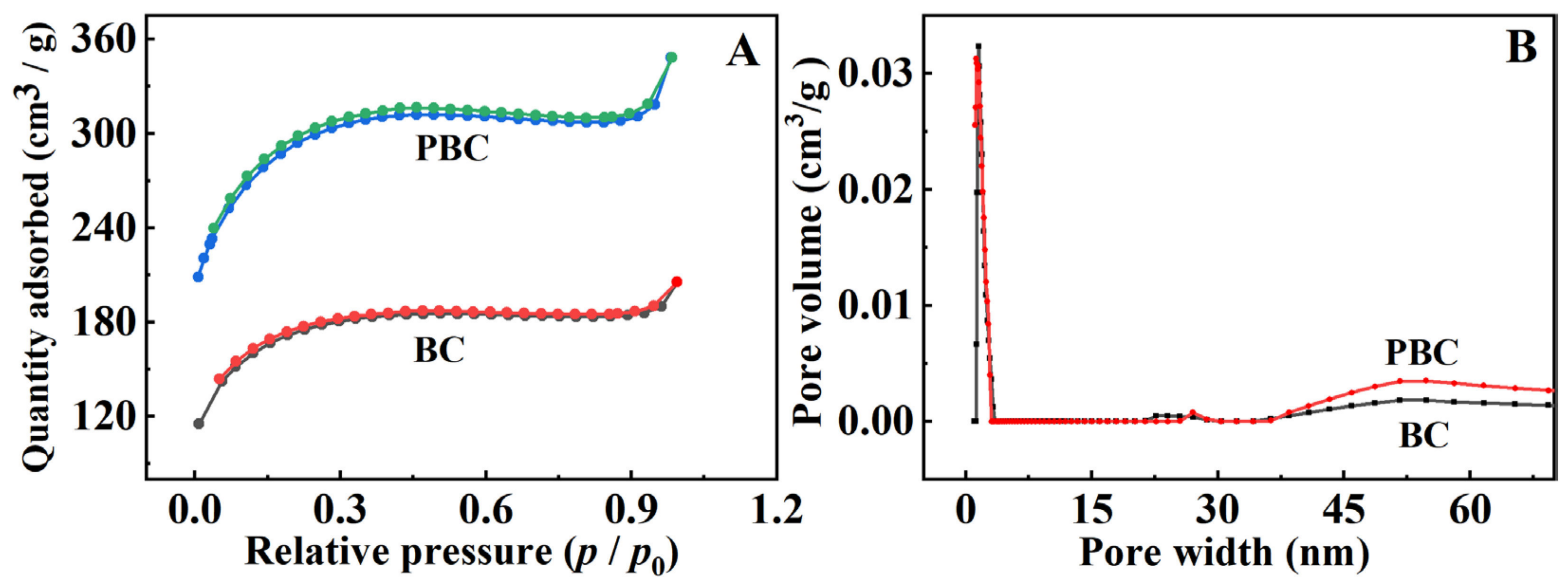
(**A**) Nitrogen adsorption/desorption isotherms and (**B**) pore size distributions of pristine BC and PBC.

The surface area and the pore structure of the synthesized pristine BC and PBC were investigated by performing a N_2_ adsorption/desorption isotherm at 77 K. As shown in Figure 3, the pristine BC sample is a typical type Ⅱ curve, demonstrating the presence of macropores. Compared with pristine BC, the BET surface area of BC increases obviously after the activation of KOH. The specific surface area of PBC is 552 m^2^·g^−1^, which is much higher than that of the pristine BC (7 m^2^·g^−1^). This phenomenon is attributed to the porous structure of PBC, which was induced via KOH etching during pyrolysis and the evaporation of volatile matter from the raw material. This result indicates that the activation of KOH promotes the development of pore structure and increases the effective surface area of BC.

### 2.2. Adsorption Studies

#### 2.2.1. Adsorption Isotherms

The adsorption isotherm of Cd^2+^ on MgZnFe-LDHs/PBC was determined by mixing 0.25 g of MgZnFe-LDHs/PBC with 25 mL of Cd^2+^ solutions of different concentrations ranging from 30 to 600 mg/L in 50 mL digestion vessels. The adsorption capacities (*Q_e_*, mg·g^−1^) were calculated using the initial concentration (*C_0_*, mg·L^−1^) of Cd^2+^ and the equilibrium concentration (*C_e_*), as well as the mass of the adsorbent (g). Equation (1) is as follows [17]:

(1) $$Q_{e}=\frac{\left(C_{0}-C_{e}\right)V}{m}$$

**Figure 4:**
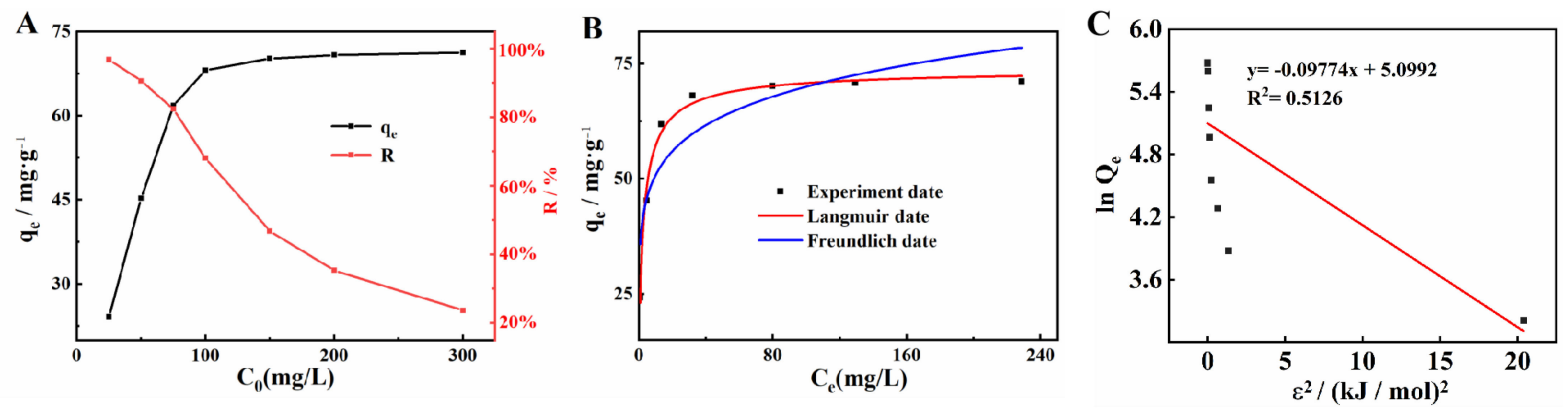
(**A**) Effect of initial Cd^2+^ concentration on Cd^2+^ adsorption; fitted (**B**) Langmuir and Freundlich, and (**C**) Dubinin–Radushkevich adsorption isotherms for Cd^2+^ on MgZnFe-LDHs/PBC.

As shown in Figure 4A, the adsorption capacity increased from 30 mg/g to 300 mg/g when the concentration of the initial solution increased from 30 mg/L to 600 mg/L. The increased adsorption capacity of MgZnFe-LDHs/PBC is caused by the increased mass transfer of Cd^2+^ between the aqueous solution and MgZnFe-LDHs/PBC at higher Cd^2+^ concentrations of the solution. The models of Langmuir [18], Freundlich [19], and Dubinin–Radushkevich [20] were used to fit the experimental data (Figure 4B,C):

(2) $$Q_{e}=\frac{Q_{m}K_{L}C_{e}}{1+K_{L}C_{e}}$$

(3) $$Q_{e}=K_{F}C_{e}^{\frac{1}{n}}$$

(4) $${InQ}_{e}={InQ}_{m}-\beta \varepsilon^{2}$$

where, *K_L_* (L/mg) is the Langmuir constant, and *K_F_* ((mg/g) (L/mg)^1/n^) is the Freundlich constant. *V* (L) and m (g) represent the volume of the Cd^2+^ solution and the mass of the MgZnFe-LDHs/PBC, respectively. 1/n is the adsorption strength, which determines the nonlinear degree of the adsorption isotherms. *ε* represents the Polanyi potential, which can be expressed as follows:

(5) $$\varepsilon =RTIn(1+\frac{1}{C_{e}})$$

*T* and *R* stand for the Kelvin temperature (K) and the ideal gas constant, respectively. The activity coefficient *β* (mol^2^·J^−2^) is related to the adsorption energy (*E*), which can be expressed as follows:

(6) $$E=\frac{1}{\sqrt{-2\beta }}$$

In general, the numerical value of *E* can be used to gain insight into the adsorption mechanism. *A* value below 8 (kJ/mol) may indicate physical adsorption; when *E* is between 8 and 16 kJ/mol, it indicates ion exchange; and chemical adsorption has been observed at an E value above 16 (kJ/mol).

According to Table 1, the high correlation coefficients (R^2^) demonstrate the agreement between the theoretical models and the experimental data. The R^2^ values are 0.98, 0.85, and 0.51 for the Langmuir, Freundlich, and Dubinin–Radushkevich models, respectively, indicating that Cd^2+^ adsorption fits the Langmuir model better than the Freundlich and Dubinin–Radushkevich models. The fit of the adsorption data to the Langmuir model indicates that the Cd^2+^ adsorption can be considered a monolayer reaction. The maximum Cd^2+^ adsorption capacity of MgZnFe-LDHs/BC is 290.3 mg/g, which is higher than that of MgFe-LDHs, ZnFe-LDHs, MgZnFe-LDHs, MgZnFe-LDHs/PBC, and other adsorbents (Table 2).

The value of the Freundlich parameter 1/n is 0.2397 and ranges from 0 to 1, indicating favorable adsorption. The value of *E* determined by the Dubinin–Radushkevich model is 2.26 kJ/mol at the experimental temperatures, indicating a process of physical adsorption. However, a very low R^2^ value (0.51) indicates that the experimental data is poorly fitted to the Dubinin–Radushkevich adsorption isotherm. Therefore, the values obtained may be outside the confidence interval [21].

**Table 2:** Comparison of adsorption capacities with other forms of adsorbent material reported in the literature for Cd^2+^.

Adsorbents	Adsorption Capacity (mg/g)	Ref.
CS ^a^/MgAl-LDH	140.8	[22]
Fe/Mn-BMBCs ^b^	138.2	[23]
HA ^c^/MgAl-LDH	155.3	[24]
Fe_3_O_4_@NiAl-LDH@guargum	258	[25]
MgFe-LDHs	183	This work
MgFe-LDHs	129.4	This work
MgZnFe-LDHs	256.6	This work
MgZnFe-LDHs/PBC	290.3	This work

Note: ^a^: chitosan; ^b^: binary metal oxide-ball-milled biochar; ^c^: humic acid.

#### 2.2.2. Adsorption Kinetics

**Figure 5:**
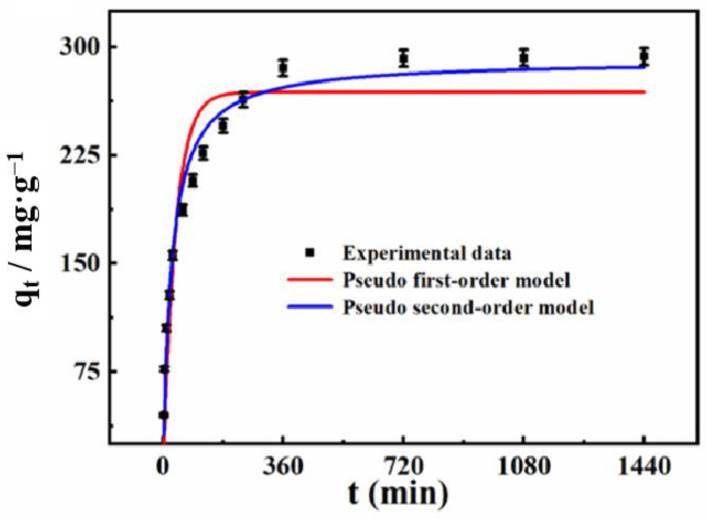
Adsorption capacity of MgZnFe-LDHs/PBC toward Cd^2+^ at different times.

To determine the adsorption rates and the appropriate contact times (0–1440 min) for the removal of Cd^2+^, the adsorption kinetics of the MgZnFe-LDHs/PBC were studied by mixing 0.25 g of the MgZnFe-LDHs/PBC with 25 mL of Cd^2+^ solutions of 300 mg·L^−1^. As shown in Figure 5, the adsorption capacity of Cd^2+^ onto MgZnFe-LDHs/PBC increases rapidly within the first 360 min, and then gradually transitions to equilibrium. The initial rapid phase is due to the numerous adsorption sites on the MgZnFe-LDHs/PBC exterior, which are associated with a high concentration of Cd^2+^ in the solution. With time, the adsorption sites of Cd^2+^ on the MgZnFe-LDHs/PBC spread from the surface to the holes, and the free Cd^2+^ can further react with these active sites. Therefore, the adsorption results of Cd^2+^ decreased relatively slowly after the equilibrium point.

To further determine the adsorption behavior of MgZnFe-LDHs/PBC toward Cd^2+^, the experimental data were fitted to the pseudo first-order (PFO) [26] and the pseudo-second-order (PSO) [27] models. The models were formulated as follows (Figure 5):

Pseudo-first order model:

(7) $$Q_{t}=Q_{e}(1-e^{-k_{1}t})$$

Pseudo-second order model:

(8) $$\frac{t}{Q_{t}}=\frac{1}{k_{1}Q_{e}^{2}}+\frac{t}{Q_{e}}Q_{e}$$

where, *Q_t_* (mg/g) represents the adsorption capacity of Cd^2+^ at time t (min). *k_1_* (min^−1^) and *k_2_* (g/(mg/min)) are the equilibrium rate constants of the PFO and PSO models, respectively. The parameters of the two kinetic models are listed in Table 3. The coefficient of determination (R^2^) values of the pseudo-first order and pseudo-second order models for MgZnFe-LDHs/PBC are 0.8847 and 0.9662, respectively. Therefore, the pseudo-second-order model might be more suitable for representing the adsorption kinetic equilibrium than pseudo-first-order model, demonstrating that the adsorption process is mainly caused by chemical adsorption.

**Figure 6:**
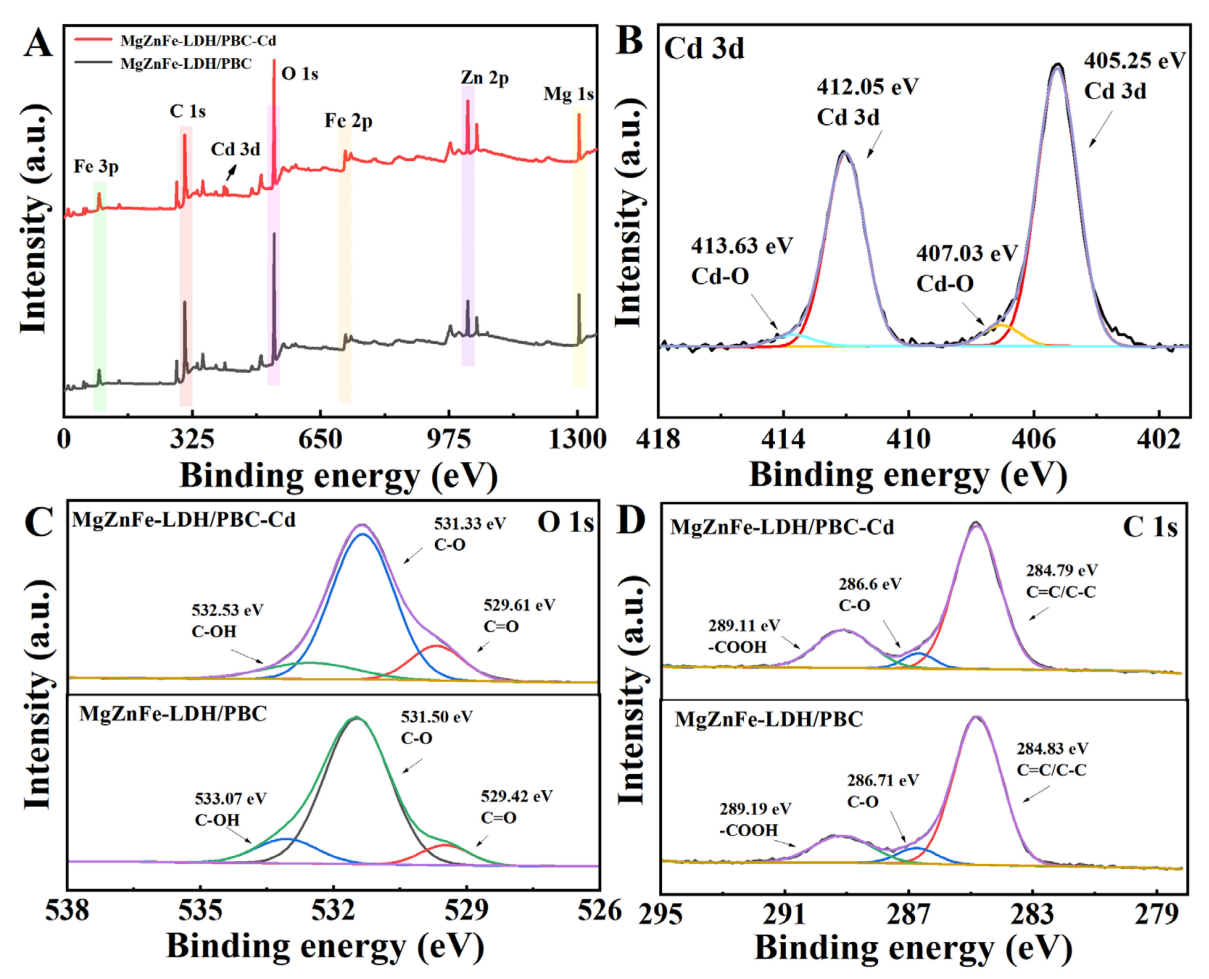
XPS spectra for MgZnFe-LDHs/PBC before and after Cd^2+^ uptake: (**A**) survey, (**B**) Cd 3d, (**C**) O 1s, and (**D**) C 1s.

To determine the adsorption mechanisms, the X-ray photoelectron spectroscopic (XPS) analyses of the surface chemical properties before and after adsorption were examined. The wide-scan spectra of MgZnFe-LDHs/PBC before and after Cd^2+^ adsorption are shown in Figure 6A. Comparison of the XPS spectra of MgZnFe-LDHs/PBC before and after adsorption shows that the Cd 3d peak appears in the XPS pattern after the adsorption of Cd^2+^ by MgZnFe-LDHs/PBC (Figure 6B). The O1s peak of MgZnFe-LDHs/BC consists of three distinct peaks at 533.07, 531.50, and 529.42 eV, indicating -OH, C-O, and C=O peaks, respectively [28] (Figure 6C). After the adsorption of Cd^2+^ by MgZnFe-LDHs/PBC, the peak areas’ C-O and -OH decreases, and the peak positions shift from 531.50 eV and 533.07 eV to 531.33 eV and 532.53 eV, respectively. This phenomenon might be due to the fact that the electron-deficient metal cation reduces the density of the electron clouds -OH and -COOH around the O atom and generates Cd-O [29,30]. The primary binding site on MgZnFe-LDHs/PBC is a functional group containing oxygen, which is involved in immobilizing heavy metal ions. XPS spectra with high-resolution of C1s are illustrated in Figure 6D. The C1s peaks of MgZnFe-LDHs/PBC can be classified as 284.83 eV, 286.71 eV, and 289.19 eV, representing C=C/C-C, C-O, and COOH, which are advantageous for surface complexation with Cd [31]. After Cd^2+^ adsorption, the C-O peaks of MgZnFe-LDHs/PBC are slightly shifted to 286.6 eV, and the C=C/C-C peaks are shifted to 289.79 eV. Moreover, the C-O content decreases from 6.71% to 5.11%, while the C=O content increases from 16.83% to 22.82%. Therefore, we can reasonably assume that C-OH is oxidized to C=O due to the reaction between C-OH and Cd^2+^ during the adsorption of Cd^2+^ by MgZnFe-LDHs/PBC.

### 2.3. Electrochemical Characterizations

**Figure 7:**
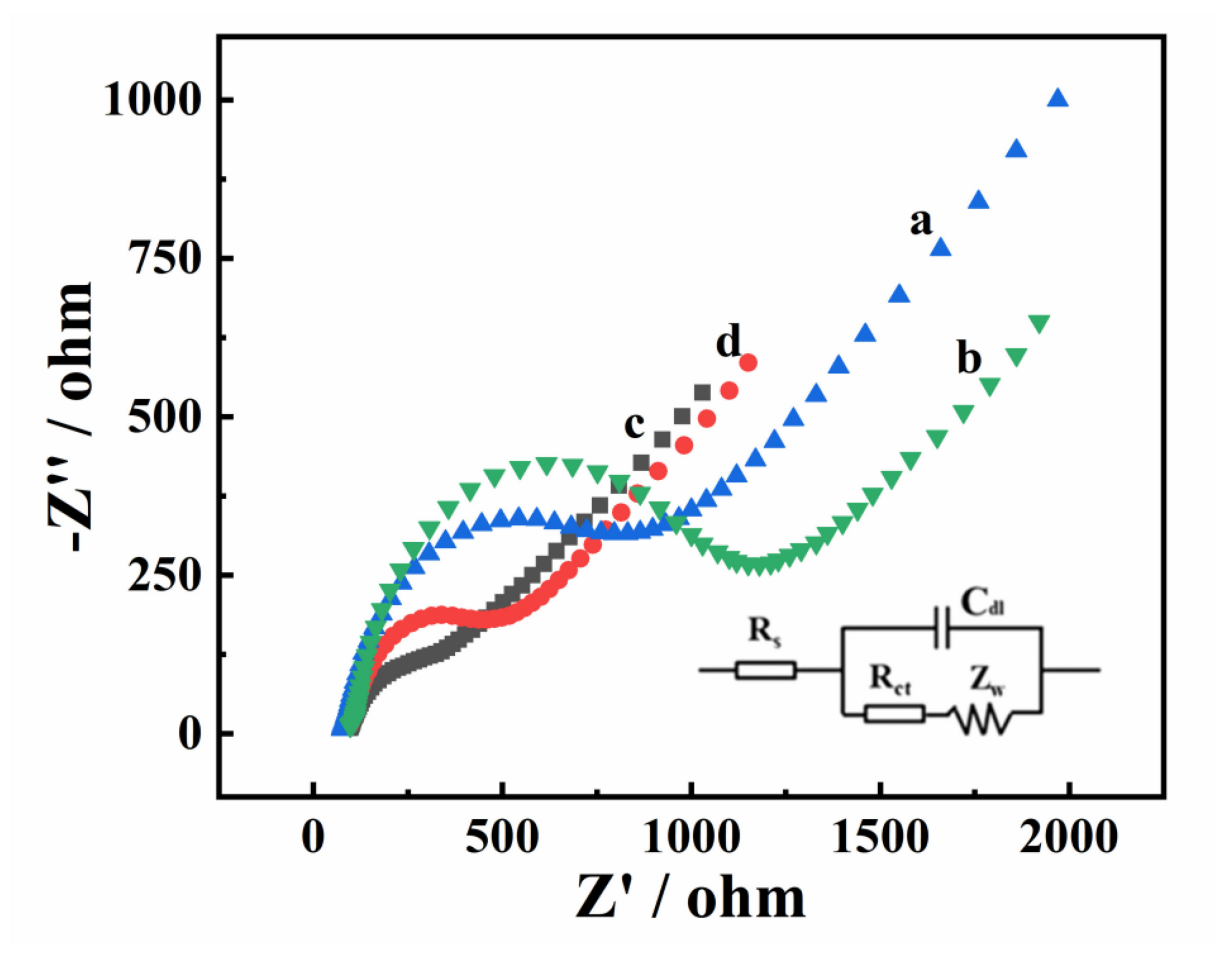
EIS of bare GCE (curve a), MgZnFe-LDHs/GCE (curve b), PBC/GCE (curve c), and MgZnFe-LDHs/PBC/GCE (curve d).

The electron transport behavior of various electrodes was investigated using electrochemical impedance spectroscopy (EIS) (Figure 7). In general, the semicircle in the high-frequency region corresponds to the charge transfer resistance (Rct), which can be estimated from the diameter of the semicircle. It can be seen that the semicircle diameter of MgZnFe-LDHs/GCE (curve b, 1076.3 Ω) is larger than that of a bare glassy carbon electrode (GCE) (curve a, 735.4 Ω), indicating that the electron transport performance of MgZnFe-LDHs is poor, which is consistent with previous reports [32]. In contrast, PBC/GCE has an Rct value of 102.7 Ω (curve c), which is due to the excellent electrical conductivity of PBC. For MgZnFe-LDHs/PBC/GCE (curve d), the Rct value (281.8 Ω) is between MgZnFe-LDHs/GCE and PBC/GCE, indicating that the combination with BC can effectively improve the conductivity of MgZnFe-LDHs, which makes MgZnFe-LDHs/PBC a potential electrode modification material.

**Figure 8:**
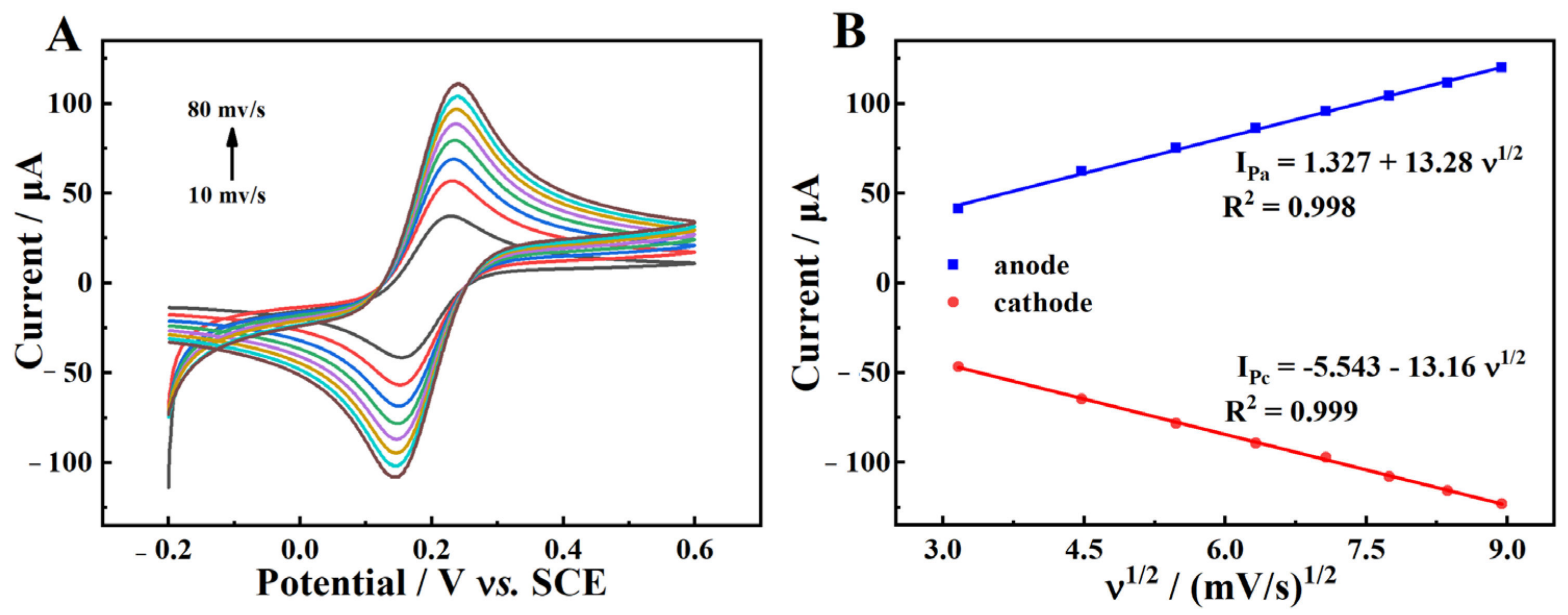
(**A**) CV curves of MgZnFe-LDHs/PBC/GCE in 0.1 M KCl solution with 5 mM [Fe(CN)_6_]^3−/4−^ at different scan rates; (**B**) the linear relationship between the peak current and v^1/2^.

To determine the effective surface area (A_eff_) of MgZnFe-LDHs/PBC/GCE, cyclic voltammetry (CV) was performed in a 0.1 M KCl solution containing 5 mM [Fe(CN)_6_]^3−/4−^ at various scan rates. The results in Figure 8A show that the peak current gradually increases with the increase of the scan rate. At the same time, a significant linear correlation is observed between the peak current and the square root of the scan rate (*v*^1/2^) (R^2^ = 0.99, Figure 8B). The Aeff of MgZnFe-LDHs/PBC/GCE is calculated to be 0.115 cm^2^ according to the Randles–Sevcik equation [33] (Equation (6)). The large surface area indicates that more adsorption sites can be exposed, so more Cd^2+^ can be enriched on the MgZnFe-LDHs/PBC/GCE surface, which gives it an excellent detection performance for Cd^2+^.

(9) Ip=2.69×105n32AeffD012c0v12

where, *I_p_* is the anodic peak current, n represents the electron-transfer number, and Aeff stands for the effective surface area of the electrode. D_0_ and *c_0_* refer to the diffusion coefficient and the concentration of [Fe(CN)_6_]^3−/4−^, respectively.

### 2.4. Electrochemical Behaviors of Cd^2+^ on Various Electrodes

**Figure 9:**
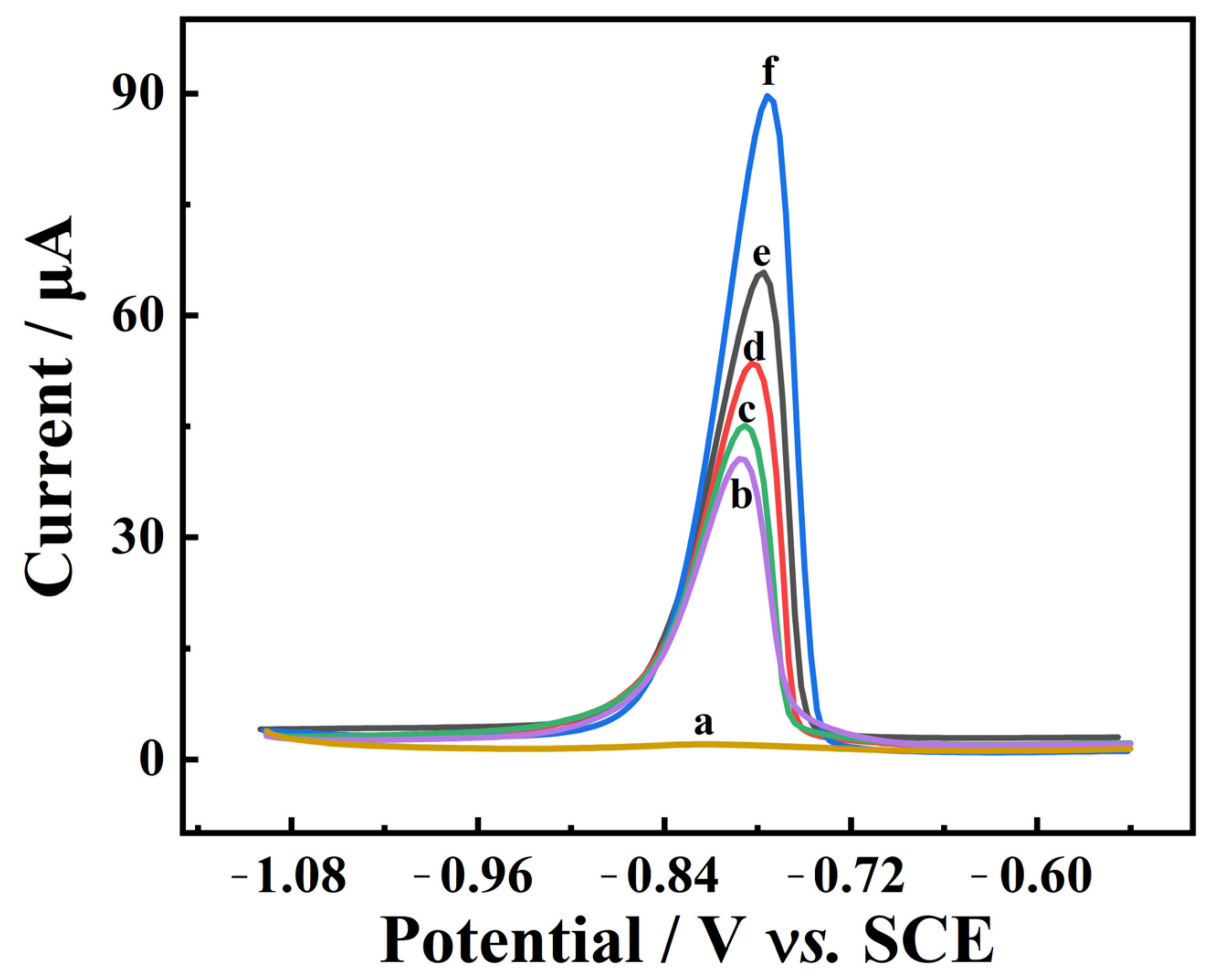
DPASV of 1 mg/L Cd^2+^ on bare GCE (curve a), MgFe-LDHs/GCE (curve b), ZnFe-LDHs/GCE (curve c), MgZnFe-LDHs/GCE (curve d), PBC/GCE (curve e), and MgZnFe-LDHs/PBC/GCE (curve f) in ABS (pH = 4.5).

The electrochemical responses of various modified electrodes for 1.0 mg/L Cd^2+^ were studied via differential pulse anodic stripping voltammetry (DPASV) in 0.1 M ABS. As can be seen in Figure 9, there is a negligible stripping peak on the bare GCE (curve a). However, significant stripping peaks of Cd^2+^ at about −0.76 V are found on the GCE modified with LDHs, indicating that these LDHs have excellent adsorption properties for Cd^2+^. Among them, the response current on MgZnFe-LDHs/GCE (curve d) is much stronger than on MgFe-LDHs/GCE (curve b) and ZnFe-LDHs/GCE (curve c), which is mainly due to the fact that more defects on the ternary LDHs’ surface expose more active sites. Similarly, a clear stripping peak with a large response current is observed on PBC/GCE (curve e), which is due to the excellent electrical conductivity and large surface area of PBC. The combination of MnZnFe-LDHs and PBC (curve f) further increases the response current, which is due to the synergistic effect between the outstanding electrical conductivity of BC and the satisfactory Cd^2+^ adsorption performance of MnZnFe-LDHs. Hence, MnZnFe-LDHs/PBC is selected as the optimal electrode modification material to construct a Cd^2+^ electrochemical platform.

### 2.5. Optimization of Experimental Parameters

In order to find the optimal conditions, a number of parameters such as the deposition potential, deposition time, and pH of ABS were studied.

**Figure 10:**
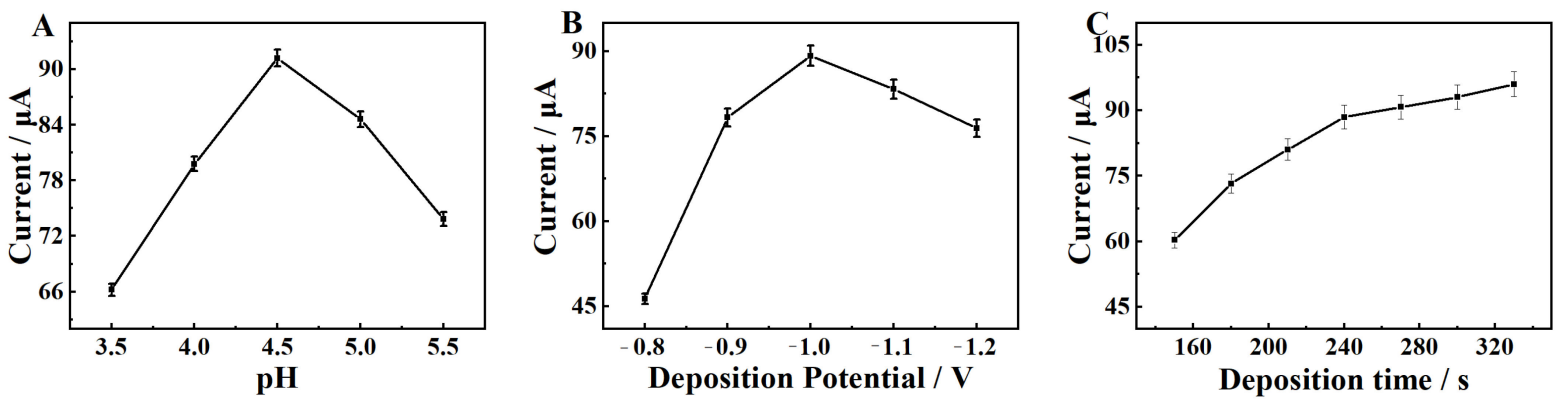
Influence of (**A**) pH value of ABS, (**B**) deposition potential, and (**C**) deposition time on the stripping peak current of Cd^2+^ on the MgZnFe-LDHs/PBC/GCE.

Considering that the pH of ABS has a great influence on the reaction current of Cd^2+^, the pH was optimized from 3.5 to 4.5. The result in Figure 10A shows that the peak current gradually increases when the pH is increased from 3.5 to 4.5. This phenomenon could be attributed to the protonation of the hydrophilic groups on the MgZnFe-LDHs/PBC/GCE surface [34] when the pH is lower than 4.5, which leads to a decrease in the Cd^2+^ adsorption sites. At a pH above 4.5, the peak current gradually decreases, which is attributed to the fact that Cd^2+^ undergoes hydrolysis at pHs above 4.5 [35], leading to a decrease in free Cd^2+^. Accordingly, 4.5 is used as the optimum pH for the following experiment.

The study of the influence of the deposition potential is shown in Figure 10B. It can be clearly seen that the peak current of Cd^2+^ increases with the negative shift of deposition potential from −0.8 V to −1.0 V. This is due to the fact that Cd^2+^ can be more completely deposited when the potential is shifted negatively [36]. However, the peak current starts to decrease when the deposition potential shifts further down, which could be caused by hydrogen evolution when the potential is too negative [37]. Therefore, −1.0 V is used as the deposition potential for the following experiment.

Figure 10C shows the effect of deposition time on the detection of Cd^2+^. As shown, the current response of Cd^2+^ increases significantly with the increase of deposition time from 150 to 240 s, indicating that more Cd^2+^ is deposited on the MgZnFe-LDHs/PBC/GCE surface. However, when the deposition time is longer than 240 s, the trend of current change is particularly slow, and gradually flattens. The phenomenon could be due to the fact that all adsorption sites for Cd^2+^ on the MgZnFe-LDHs/PBC/GCE surface are occupied [38]. Therefore, 240 s was chosen as the optimum deposition time for the experiment.

### 2.6. Electrochemical Detection of Cd^2+^ with MgZnFe-LDHs/PBC/GCE

**Figure 11:**
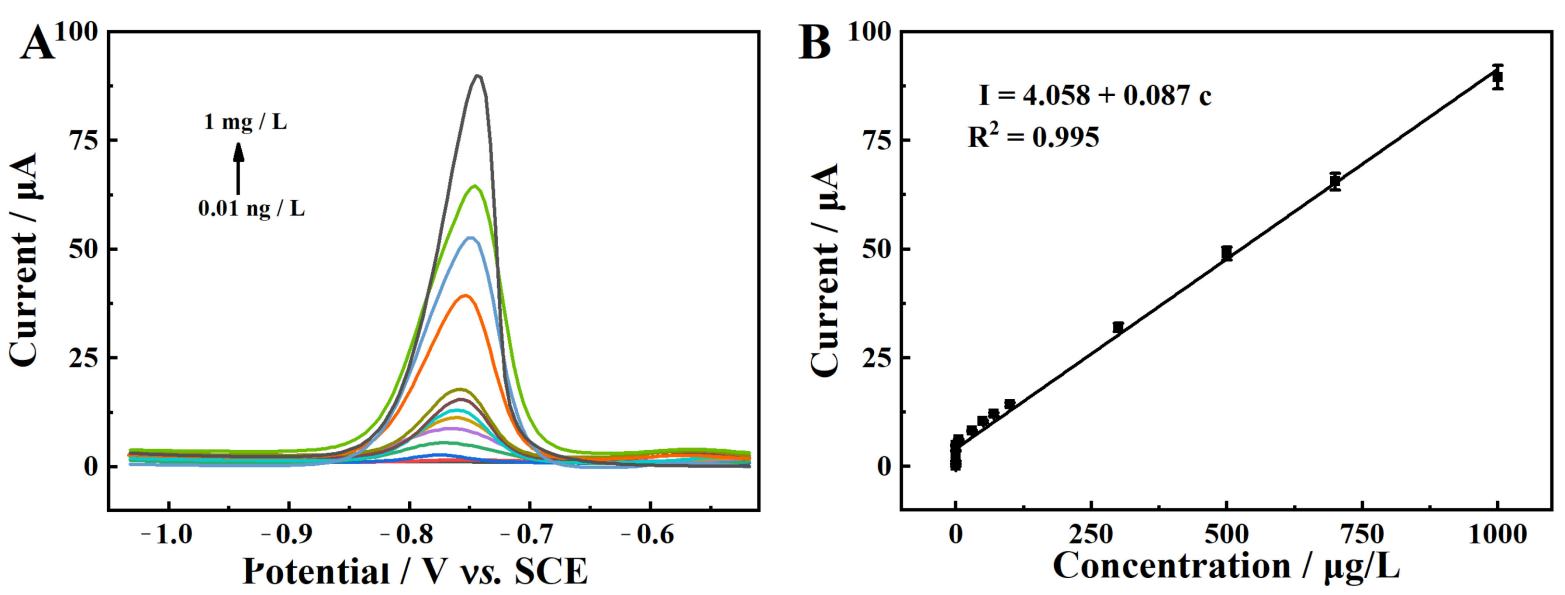
(**A**) DPASV curves of MgZnFe-LDHs/PBC/GCE in ABS (pH = 4.5) with different Cd^2+^ concentrations; (**B**) linear relationship between peak current and Cd^2+^ concentration.

The electrochemical sensing performance of MgZnFe-LDHs/PBC/GCE with regard to Cd^2+^ was investigated via DPASV under optimized conditions. The results in Figure 11A show that the peak current of Cd^2+^ at −0.76 V increases with the addition of Cd^2+^. Moreover, there is a good linear relationship between the peak current and Cd^2+^ concentration in the range of 0.01 ng/L to 1 mg/L (Figure 11B). The regression equation is expressed as I = 0.087c + 4.058 (R^2^= 0.99). Accordingly, the detection limit (LOD) of the Cd^2+^ sensor is calculated to be 3.0 pg/L (S/N = 3), which is much lower than the approved Cd^2+^ concentration for drinking water (1300 μg/L).

### 2.7. Repeatability, Reproducibility, and Selectivity

**Figure 12:**
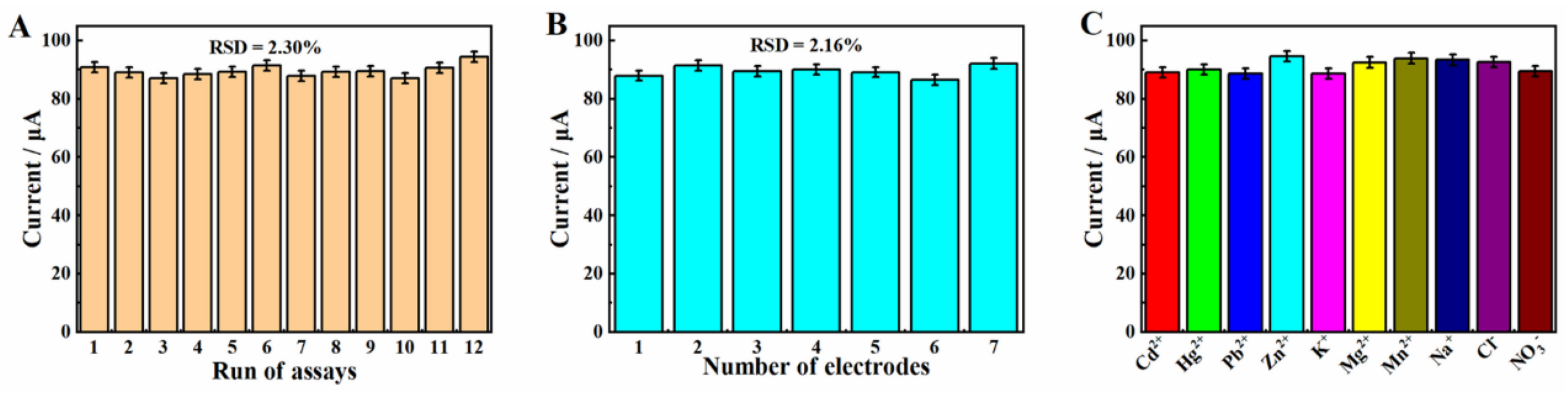
(**A**) Twelve consecutive measurement results of MgZnFe-LDHs/PBC/GCE versus 1.0 mg/L Cd^2+^; (**B**) current response of seven independent MgZnFe-LDHs/PBC/GCEs versus 1.0 mg/L Cd^2+^; (**C**) current response of MgZnFe-LDHs/PBC/GCE versus 1.0 mg/L Cd^2+^ in the presence of 50-fold concerted interferers.

First, a MgZnFe-LDHs/PBC/GCE was used to detect 1.0 mg/L Cd^2+^ twelve times consecutively to investigate the repeatability of the proposed sensor. The relative standard deviation (RSD) of the twelve measurement results is 2.30% (Figure 12A), demonstrating the excellent repeatability of the MgZnFe-LDHs/PBC/GCE. In addition, seven parallel MgZnFe-LDHs/PBC/GCEs were used to detect 1.0 mg/L Cd^2+^ under the same conditions to investigate the reproducibility. Figure 12B shows that the current response of the seven modified electrodes is nearly flat with an RSD of 2.16%, indicating the excellent reproducibility of MgZnFe-LDHs/PBC/GCE for Cd^2+^.

The selectivity of MgZnFe-LDHs/PBC/GCE toward Cd^2+^ was also investigated. Several common interfering ions were each added to a 1.0 mg/L Cd^2+^ solution. The current response was measured via the DPASV method using MgZnFe-LDHs/PBC/GCE. As shown in Figure 12C, at 50 mg/L, various interfering ions such as Hg^2+^, Pb^2+^, Zn^2+^, K^+^, Mg^2+^, Mn^2+^, Na^+^, Cl^−^, and NO_3_^−^, were insignificant on the response current of the 1.0 mg/L Cd^2+^, demonstrating the excellent selectivity of the electrochemical sensor based on MgZnFe-LDHs/PBC/GCE for Cd^2+^.

### 2.8. Practical Application

To verify the practicality of this sensor, MgZnFe-LDHs/PBC/GCE was used to detect Cd^2+^ in a paddy water sample, collected from Jiangxi Agricultural University (Nanchang, China). The collected paddy water was first filtered with a 0.45 µm filter membrane, and then diluted with 0.1 M ABS. Then, a Cd^2+^ standard solution with different concentrations was added to the treated paddy water samples, and a recovery study was carried out to investigate the sensing ability of MgZnFe-LDHs/PBC/GCE for Cd^2+^ in real samples. As shown in Table 4, the good recovery (98.80–104.0%) and RSD (2.2–3.9%) indicate the satisfactory practical capability of MgZnFe-LDHs/PBC/GCE in Cd^2+^ detection.

## 3. Experimental Section

### 3.1. Reagents and Instruments

Rape stalks were obtained from Jiangxi Agricultural University. Mg(NO_3_)_2_, Zn(NO_3_)_2_, Fe(NO_3_)_3_, Cd(NO_3_)_2_, NaOH, and Na_2_CO_3_ were purchased from Beijing Chemical Industry CO., Ltd. 0.1 M acetate buffer solution (ABS) was prepared with 0.1 M NaAc and HAc. All reagents and solvents were used directly without further purification.

**Scheme 1:**
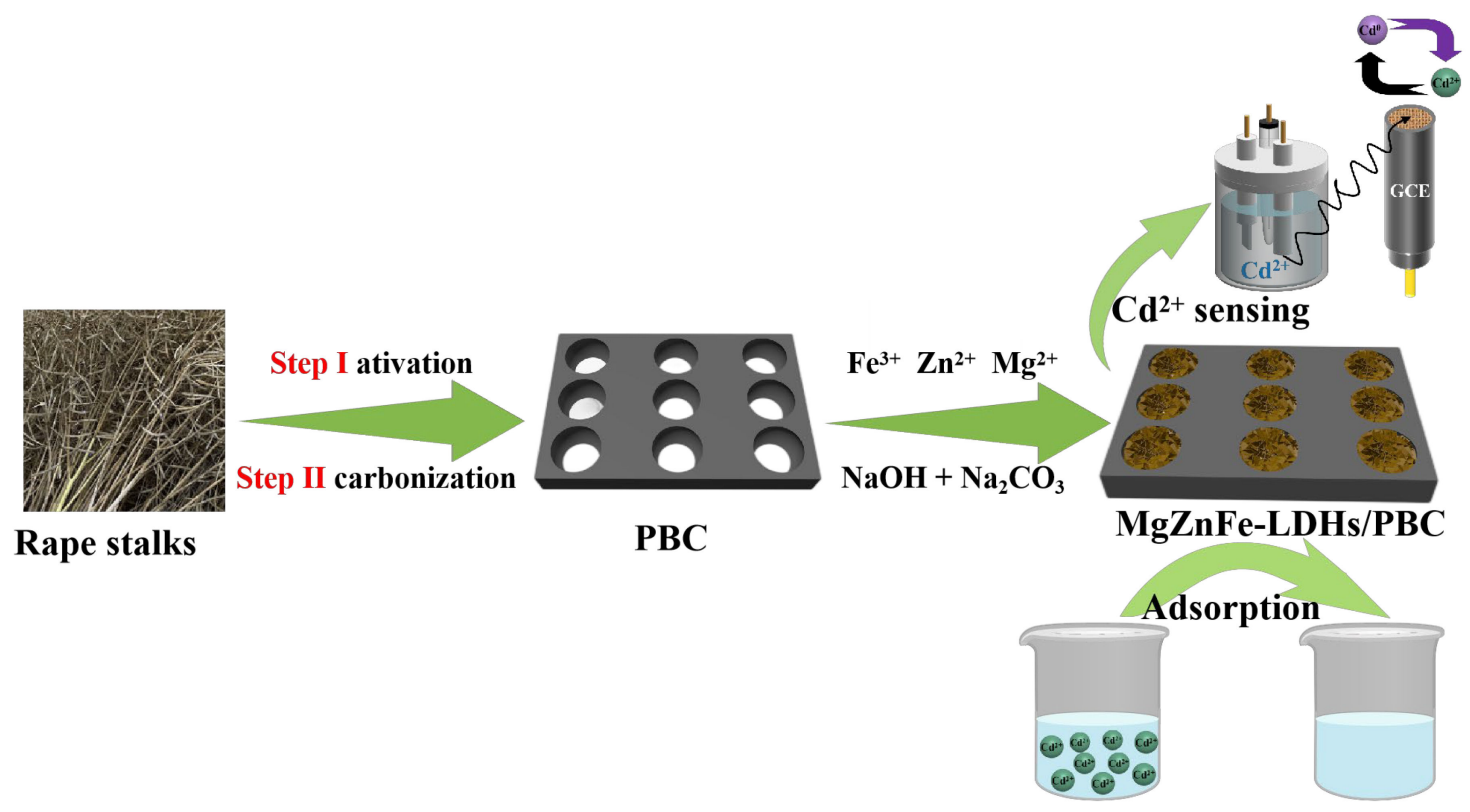
Preparation of MgZnFe-LDHs/PBC composite and its application for the adsorption and detection of Cd^2+^ in aqueous solution.

Surface morphology was determined using Sigma 300 SEM (Hitachi S-4800, Tokyo, Japan). XRD patterns were examined in a range between 5° and 85° using a Rigaku Smart Lab X-ray diffractometer (Smartlab SE, Tokyo, Japan). XPS measurements were performed using a Thermo Fischer ESCALAB Xi+ X-ray photoelectron spectrometer (Alα hν = 1486.6 eV). All electrochemical measurements were performed on a CHI760E electrochemical workstation with platinum sheet as the counter electrode, saturated calomel electrode (SCE) as the reference electrode, and various working electrodes. Differential pulse anodic stripping voltammetry (DPASV) was employed as the detection technique. The adsorption and detection process of Cd^2+^ is listed in Scheme 1.

### 3.2. Preparation of MgZnFe-LDHs/PBC

The MgZnFe-LDHs/PBC composite was prepared using a simple co-precipitation method. In this process, the activated PBC was prepared using a previous method [39]. BC from rape stalks was prepared via slow pyrolysis of the starting material under N_2_ protection. The surface of the rape stalks was cleaned and washed several times with distilled water to remove dust and some inorganic impurities. Then, the dried rape stalks were impregnated with KOH and dried in an electric oven. The resulting product was placed in a tube furnace, and heat-treated for 2 h under an N_2_ atmosphere at a rate of 5 °C· min^−1^ at 800 °C. After cooling to room temperature, 1 mol/L HCl was added to the residue for 1 h followed by filtration and washing to neutrality. After drying at 80 °C for 8 h, PBC was obtained.

MgZnFe-LDHs/PBC were synthesized via the co-precipitation method. BC, Mg(NO_3_)_2_, Zn(NO_3_)_2_, and Fe(NO_3_)_3_ were dissolved in 100 mL of deionized water with the molar ratio of bivalent metals to trivalent metals (Mg^2+^ + Zn^2+^/Fe^3+^) fixed at 2 and Mg^2+^ to Zn^2+^ at 3. Then, 100 mL of mixed alkaline solution of 0.85 M NaOH and 0.32 M Na_2_CO_3_ was added to the above solution. Then, the mixed solution was stirred for 4 h and aged for 20 h. The temperature was maintained at 70 °C. Finally, the product was washed three times with ultrapure water and dried at 60 °C. In addition, MgZnFe-LDHs were prepared via the same procedure without PBC. MgFe-LDHs and ZnFe-LDHs were synthesized without Zn^2+^ and Mg^2+^, respectively.

### 3.3. Preparation of Modified Electrode

First, the glassy carbon electrode (GCE) was polished with 0.05 mm and 0.3 mm alumina slurry. Then, it was ultrasonically cleaned with absolute alcohol and distilled water. Then, 5.0 μL of MgZnFe-LDHs/PBC dispersion (1 mg/mL) was dropped on the GCE surface and dried in a room to obtain MgZnFe-LDHs/PBC/GCE. MgZnFe-LDHs/GCE and BC/GCE were prepared using a similar method by replacing MgZnFe-LDHs/PBC with MgZnFe-LDHs and PBC.

## 4. Conclusions

In this work, a low-cost but practical MgZnFe-LDHs/PBC composite was proposed for sensitive electrochemical detection and the adsorption removal of Cd^2+^. The growth of MgZnFe-LDHs in the pores of PBC significantly reduces the accumulation of MgZnFe-LDHs. The MgZnFe-LDHs/PBC composite has a large specific surface area, abundant interlayer anions and surface hydroxyl groups, which provide ample space for Cd^2+^ adsorption. The maximum adsorption capacity can reach 293.4 mg/g. For the electrochemical sensing of Cd^2+^, the MgZnFe-LDHs with their excellent Cd^2+^ enrichment performance combined with BC with its good electrical conductivity endow the electrochemical Cd^2+^ sensor with a low detection limit (3.0 pg/L), good reproducibility, stability, and selectivity. All in all, the synergistic effect between MgZnFe-LDHs and PBC makes MgZnFe-LDHs/PBC a promising material for the simultaneous electrochemical detection and removal of Cd^2+^.

## Figures and Tables

**Table 1:** Adsorption parameters of Cd^2+^ on MgZnFe-LDHs/PBC material determined by Langmuir, Freundlich, and Dubinin–Radushkevich models.

Langmuir			Freundlich			Dubinin–RadushKevich	
q_e_ (mg/g)	K_1_ (L/mg)	R^2^	1/n	K_L_ (mg/g)	R^2^	β (mol^2^·J^−2^)	R^2^
290.3	0.1284	0.9811	0.2397	84.42	0.8463	−0.09744	0.5126

**Table 3:** Adsorption kinetics parameters for the adsorption of Cd^2+^ onto MgZnFe-LDHs/PBC.

	Pseudo First-Order Model			Pseudo Second-Order Model		
*q_e_*, Exp (mg/g)	*k_1_* (min^−1^)	R^2^	*q_e_* (mg/g)	*k_2_* (g/mg·min)	R^2^	*q_e_* (mg/g)
293.4	0.0273	0.8847	268.6	0.000137	0.9662	290.8

**Table 4:** Recoveries of trace Pb^2+^ in local paddy water sample (*n* = 3).

Sample	Added (μg·L^−1^)	Found (μg·L^−1^)	RSD (%)	Recovery (%)
1	0	-	-	-
2	0.05	0.051 ± 0.004	102.0	3.9
3	0.50	0.52 ± 0.017	104.0	3.4
4	5.00	4.94 ± 0.26	98.80	2.2
5	50.00	49.68 ± 1.49	99.36	2.8

## Data Availability

The data presented in this study are available in the article.
